# Can Computers Become Conscious and Overcome Humans?

**DOI:** 10.3389/frobt.2018.00121

**Published:** 2018-10-26

**Authors:** Camilo Miguel Signorelli

**Affiliations:** ^1^Department of Computer Science, University of Oxford, Oxford, United Kingdom; ^2^Cognitive Neuroimaging Unit, INSERM U992, NeuroSpin, Gif-sur-Yvette, France; ^3^Centre for Brain and Cognition, Pompeu Fabra University, Barcelona, Spain

**Keywords:** artificial intelligence, information processing, cognitive computing, type of cognition, super machine, conscious machine, consciousness

## Abstract

The idea of machines overcoming humans can be intrinsically related to conscious machines. Surpassing humans would mean replicating, reaching and exceeding key distinctive properties of human beings, for example, high-level cognition associated with conscious perception. However, can computers be compared with humans? Can computers become conscious? Can computers outstrip human capabilities? These are paradoxical and controversial questions, particularly because there are many hidden assumptions and misconceptions about the understanding of the brain. In this sense, it is necessary to first explore these assumptions and then suggest how the specific information processing of brains would be replicated by machines. Therefore, this article will discuss a subset of human capabilities and the connection with conscious behavior, secondly, a prototype theory of consciousness will be explored and machines will be classified according to this framework. Finally, this analysis will show the paradoxical conclusion that trying to achieve conscious machines to beat humans implies that computers will never completely exceed human capabilities, or if the computer were to do it, the machine should not be considered a computer anymore.

## Introduction

During many centuries, scientists and philosophers have been debating about the nature of the brain and its relation with the mind, based on the premise of an intrinsic dualism, typically called mind-body problem (Searle, 1990; Chalmers, 1995). Arguments take one form or another, however, most of them can be reduced to one kind of dualist or non-dualist view (Lycan and Dennett, 1993). The importance of these debates acquires even more relevance when the question is stated as the possibility to build machines which would be able to reproduce some human capabilities such as emotion, subjective experiences, or even consciousness.

The problem is exacerbated when some scientists claim a new future generation of computers, machines and/or robots which would additionally overcome human capabilities. In the view of the author, these claims are based on misconceptions and reductionism of current most important issues. The idea, however, is not discarded here and is expressed, trying to avoid reductionism, in a different way to show its paradoxical consequences (Signorelli, 2018). For example, the idea of reaching and overtaking human capabilities implies the knowledge of a set of distinctive processes and characteristics which define being a human (e.g., intelligence, language, abstract thinking, the creation of art and music, emotions and physical abilities, among others). This simple idea leads to some fundamental issues. First, claims about new futurist robots do not define this set of distinctions; they do not care about the importance of what it is to be a human, what is necessary to build conscious machines or its implications. Secondly, they assume a materialist view of these distinctions (i.e., these distinctions emerge from the physical and reproducible interaction of matter) without explaining the most fundamental questions about the matter (Frank, 2017). Thirdly, they do not explain how subjective experience or emotions could emerge from the theory of computation that they assume as a framework to build machines, which will reach consciousness and overcome humans. In other words, these views do not explain foundations of computation that support or reject the idea of high-level cognitive computers. Finally, engineering challenges of building these kinds of machines are not trivial, and futurists assume reverse engineering as the best tool to deal with this when even some neuroscience techniques do not seem to give us any information about simple computing devices such as microprocessors (Jonas and Kording, 2017). Actually, if methods of neuroscience are not inferring useful information from microprocessors, it is possible to conclude that either the neurons are not working as computers or all the information that we know about cells and neurons, using these techniques, is wrong. The first option discards reverse engineering as a feasible tool to understand the brain, and the second option discards findings in neuroscience related to mechanistic and computational interpretation. Thus, it is still necessary to focus on many intermediate and fundamental steps before declaring that some computers would reach or even exceed human capabilities.

This work does not expect to solve these issues; on the contrary, the aim of this paper is to expand previous works (Signorelli, 2018) and illustrate misconceptions and misunderstanding of some crucial concepts. For example, the issue of overcoming human capabilities will be discussed in parallel with the issue of producing conscious machines, to show their close relation and same paradoxical consequences. Additionally, the importance of new concepts and ideas will be approached in a preliminary and speculative way, with the intention of developing them in further works. Following this framework in order to make clear some of the questions above, the second section will define what will be understood by human capabilities and human intelligence; the third section will confront current common views of computation, cognitive computing, and information processing; the fourth section will discuss consciousness as a basic requirement to make computers with similar human intelligence; the next two section will show a new hypothesis of how consciousness could work; then, machines will be classified in four categories based on four types of cognitions derived from consciousness requirement, and finally, according to these classifications, the last section will show some paradoxes and implications, which emerge from the idea to make machines-like-brains reaching consciousness and overcoming humans.

## A sub set of human capabilities

Usually, it is considered that computers, machines and/or robots will eventually reach, or even overtake human intelligence. This idea is supported by many advances in Artificial Intelligence (AI). For example, consecutive victories of DeepMind project vs. the GO human champion (Silver et al., 2016), or robots that have passed some kind of Self-Consciousness test (Bringsjord et al., 2015). Science fiction, movies, and writers also stimulate and play enough with the notion of “Singularity,” the precise moment where machines exceed human capabilities (Good, 1965). In this scenario, a computer/machine is called Super Machine.

Nevertheless, how much does scientific evidence support this idea? What does overcoming human intelligence mean? What does human intelligence mean? And what is the relation with consciousness? Computers already exceed human algorithmic calculations, among many others. A clear example is the recent report of AlphaGo zero which can learn without human intervention and play at super-human level (Silver et al., 2017). In fact, one option to overcome human abilities might be a cognitive system completely different to the anthropocentric science fiction view. As will be shown later, this kind of computer may reach and overcome some, but not all, human capabilities. That is why; one position could claim that it is not necessary to assume computers like brains or conscious machines to overtake human capabilities. It is a valid point; however, will this kind of computer surpass human brain only in a rational/algorithmic way or also an emotional one? Will this kind of computer be able to dance better than us, to create better than us, to feel better and like us? Otherwise, it will never reach nor overtake human abilities. One reason is that part of being human is to have emotional behavior, to be able to dance, create, etc, additionally to our apparently rational behavior. As it was mentioned above, the first issue emerges: what does human being mean? If what is being a human and which abilities need to be overcome are not understood, how can we ever think about overcoming unknown capabilities? For example, human intelligence may not be only associated with logical, algorithmic, or rational thinking. Types of intelligence have already been suggested, which are closely related to each other such as kinaesthetic and emotional intelligence in humans (Sternberg, 1997; Gardner, 1999). So far, implementing emotions or simple movements in machines is equal to or more complicated than implementing rational or algorithmic intelligence (Moravec, 1988). Actually, current implementations of emotions in machines are based on a logical, computable and deterministic approaches, leaving out essential characteristics of emotions such as that emotions interfere with rational processes and optimal decisions. In fact, these implementations are founded on the idea that emotions play an important role in making humans more efficient, rationally speaking (Martinez-Miranda and Aldea, 2005), when cognitive fallacies are showing the contrary (Gilovich et al., 2002; Kahneman, 2003) and experiments on neuroscience from the called default neural network, which is related to self-oriented information, are suggesting anti-correlated subsystems of information processing (Simpson et al., 2001; Fox et al., 2005; Buckner et al., 2008) which interfere each other. The view of computer non-like-brain does not care about these issues and assumes intelligence as only rational, logic and computable capability; or even worst, the problem of computer non-like-brains defenders is to think that some properties of life could be replicated without the distinctive properties of being alive.

Then, is it possible to define a set of human characteristics? Futurists assume the existence of this set but they do not define it in any way. While any serious attempt to define a human set or a subset should first start with a definition of living entities. One possible definition is the notion of autopoiesis (Maturana and Varela, 1998) which refers to the self-reproduction and self-maintenance of a system. In this view, a living machine is a unitary system or network of processes which is able to regenerate through their interactions and continuous transformation. Even when it is still controversial a complete definition of living beings and the utility of the autopoiesis concept (Fleischaker, 1992), two characteristics, autonomy and reproduction, emerge as key features of living beings. Some critics of this concept state that autopoiesis does not consider external references that can be crucial for the organism. Therefore, a probable better definition of the living being may be a unitary system or network of processes which interacts with the environment to keep their autonomy and increase their capability to reproduce. Of course, any definition of life is a huge enterprise and the goal of this essay is not to answer this question, but state a simple and probably the simplest definition that can help us to decide when a machine reaches and overcomes human characteristics. Interestingly, this general definition does not discard the idea that other systems or machines can reach these two characteristics, even when they should not be considered living machines. In fact, this is not contradictory because autonomy and reproduction are thought here as a subset of living machine properties; it means that they are necessary conditions but not sufficient to be considered living machines. Thus, humans, as well as other animals, are autonomous entities with the ability to reproduce.

Additionally, however, it is also necessary to identify at least one characteristic to differentiate human being from other living beings. One historical proposal has been the notion of morality. Morality and ethics can be understood as high-level reasoning to distinguish between proper or improper behavior and intentions. This notion also implies a community, a culture and social obligations within that community. Morality has been studied by many philosophers as for example (Hegel, 2001) and (Kant, 1785), and connected with concepts as rationality, free will, and consciousness. Nevertheless, when neuroscientists look for correlates or building blocks of morality inside of the brain, it is possible to find areas which are associated with empathy and social interaction, mostly identified with emotional states (Bzdok et al., 2012). In these terms, morality is not only a rational process as some philosophers proposed (Kant, 1785), and it is apparently not exclusive of human beings. Thus, the notion of a uniquely human characteristic remains too elusive and what it is necessary to explain and replicate in robots is still not clear (Chappell and Sloman, 2007). That is why; the suggestion in this work is to define human morality as a complex process where rational and emotional thinking takes part, then, moral decisions, moral behavior, and moral intentions emerge only after this intricate process takes place. In other words, the distinctive ingredient in human intelligence will be considered the capability to integrate rational and emotional thinking to take moral decisions which are adapted to the context. It is not clear that animals can integrate, as a whole, rational thinking and emotional thinking to take moral decisions, however, even if some animals could be able to do it, the assumption here is that the kind of morality emerged would be different and characteristic of each species, culture and even subjects. In other words, as it will be shown later, morality is a complex behavior intrinsically related to context, subjectivity, and consciousness.

The definition of a general intelligence can also be inferred from the previous discussion, at least in a preliminary way. It is interesting to point out that a general definition of intelligence and human intelligence is still a question of debate, since the pioneering works of Turing (Turing, 1950) until our days, where the definition changes according to how science and AI evolve (Nilsson, 2009; Stone et al., 2016). Nevertheless, based on previous comments, general intelligence can be understood as the capability of any system to take advantage of their environment to achieve a goal. Biologically speaking this goal is maintaining the autonomy and reproduction, that is to say: survive; while the goal in machines can be solving a specific task or problem using internal and external resources. This general definition can incorporate living beings as well as robots and computers, and in this way, intelligence is general enough to include different kinds of intelligence, contextual influences and different kind of systems with different degrees of intelligence. Finally, also in these terms, human intelligence would be the ability to take advantage of their social environment to keep autonomy and reproduction thanks to a balance between rational and emotional information processing. This human intelligence definition incorporates the set of distinctive characteristics which define partially being human, and where the advantage can take place through cognition, learning, memory; among other processes needed to achieve the goal.

**Figure 1:**
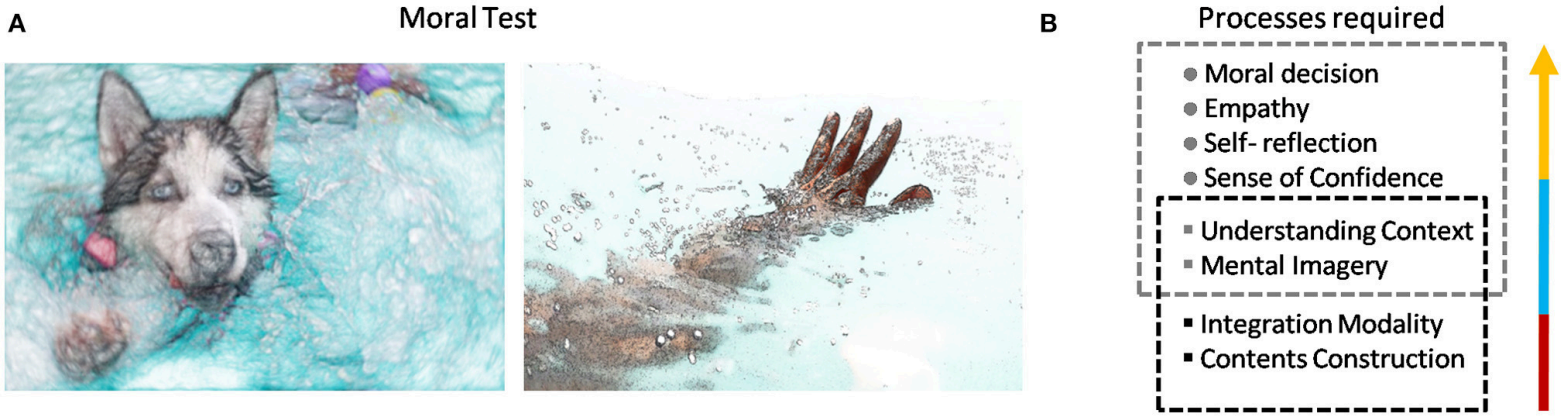
Moral Test and Processes required. **(A)** Moral test and moral dilemmas are suggested to test when a machine has reached human kind of thinking. **(B)** Some processes required for moral thought are stated as examples, among many other possible processes needed.

At this point is inevitable to shortly mention something about potential tests to prove if a machine reached or not the criteria of human intelligence. Turing was the first one to suggest a test based on a simple exchange of words, questions and answers (Turing, 1950). In its simplest version, this exchange is between a machine and a human who should decide if the machine is a machine or another human. The test is simple, in the sense of its simple execution, and at the same time complex, in the sense that it should capture as many as possible features of the human being. Turing probably realized that the complexity of human intelligence was not only associated with rational and logical processes. That remains evident in the way as he proposed his test as a simple written conversation and also when he refers to the incorporation of human mistakes in future machines to be able to pass the test. However, the Turing test has been criticized many times, where the main against argument is summarized by Searle in his Chinese room example (Searle, 1980). A full review of this topic would be part of an entirely new document and indeed, it will be part of further works. In this way and from the definition of human intelligence stated above, it seems better to suggest a test founded on moral dilemmas more than simple day to day questions (Signorelli and Arsiwalla, 2018). Moral dilemmas are simple, in the sense that they do not require any kind of specific knowledge, but at the same time very complex even for humans, because some of them require a deep understanding of each situation, and deep reflexion to balance moral consequences, emotions, and optimal solutions. No answer is completely correct, they are context dependent, and solutions can vary among cultures, subjects, or even across the same subject in particular emotional circumstances. In other words, a moral test, grounded on moral thinking, needs intermediate processes which are characteristics of high-level cognition in human, as for example self-reflection, sense of confidence and empathy, among others. Hence, a machine will reach part of what it is defined as human intelligence if the machine is able to show autonomously speaking the intricate type of thinking that humans have when they are confronted to these kinds of dilemmas. To do that, it is necessary to focus on intermediate steps reaching some of the previous processes of moral thinking in humans (Figure 1).

One example of a moral test is the next situation (Figure 1A): If you are in an “emergency boat” after a shipwreck and the boat has only one space left, who would you admit to in the boat and why: a big, healthy and young dog or an injured and sick old man? The answer is not obvious and actually, it is one of the most debated topics in biomedical research, because it does not involve only human morality but also inter-species issues on animal experimentation. What could be the answer of a machine to this question? What could be the logical and emotional thinking of this machine? What is, in fact, the answer of the reader? There are very good reasons to take any of both possible decisions, even a third and fourth answer is also possible, however, the important point is the way how to reach to a conclusion and not the conclusion itself. Of course, many critics should be addressed before to claim that a moral test would be a good test to capture the machine intelligence, compared with human intelligence. For example, according to what types of answers will the comparison be made? What would happen if the machine develops its own sense of morality? Will we be able to recognize it? Tests for machines apparently make sense only when it is desirable to compare them with human intelligence, but in fact, if the machine reaches consciousness, it is also possible that the machine develops a new kind of morality based on non-anthropocentric views and even new possible answers to many moral dilemmas.

For the purpose of this work, we will need to assume that there is a certain set of “human being” properties formed by at least a subset of three features: Autonomy, Reproduction, and Morality. Therefore, it is possible to decide when an animal or machine reach or not the condition to be part of this set, even though it is known that the definition of this set is one of the most controversial and debated issues. Moreover, to reach these three main elements it is necessary to incorporate many intermediate steps and some of them will be discussed in next sections. For example, robots and computers are rarely autonomous in the biological sense; they definitely cannot replicate, re-structure or even recover from harm by themselves. However, these issues can be overcome in the future, at least in a functional way. The only huge issue that is not possible to implement without a deeper understanding of human beings is the morality question, paradoxically, an important distinctive human characteristic, closely to human intelligence and consciousness. Morality requires many previous processes usually considered as high-level cognition, starting with decision-making to self-reflection, to be able to detect mistakes on these decisions; sense of confidence, to estimate how correct a decision or action is; mental imagery, to create new probable scenarios of action; empathy, to equilibrate individual and social requirements; understanding of context, to adapt moral decisions to the context, among others. Because these processes are sharply connected with consciousness, as it will be shown in next sections, a moral test is also a kind of consciousness test. Until now, brains are the only types of systems that have these processes and focusing on how they are working will help us to understand what it would be necessary to replicate in robots for them to reach consciousness and potentially achieve high-level cognition.

Further work and potential experiments can be influenced by these preliminary ideas, in order to improve the behavior of robots/machines trying to answer what is necessary to replicate a truly moral behavior in them.

## Information processing in the brain

One supporting fact about the idea of reaching consciousness and overcoming human capabilities with computers comes from the exponential increase of computational capacity or Moore's law (Moore, 1998). This increase should impact on the development of new technologies until reaching intelligence levels of the human brain. Beyond this view, there is the assumption that the brain works as a computer and its processing could work by analogy with computational processes. Of course, the brain is a physical entity as computers are; it partially works with electrical signals, resolves complex problems and is processing information in one way or another. Nevertheless, the way the brain processes information is still unknown and, it may not be a digital computation, or rather not be information processing in computational abstract terms at all (Epstein, 2016). Information processing implies processes where input are changed to become outputs; however the brain could be working in a new regime, where the distinction between inputs and outputs could not exist, even causalities could be completely different to what we know until now. In this context, it should be possible to speak about another kind of processing as “replication processing,” “simulations” (Arsiwalla et al., 2018) or maybe “abstract models,” which could be self-informative to some singular physical systems like brains. It is also known that brains work with complex neuromodulation (Nusbaum et al., 2001), stores information in a sparse and unknown way (Tetzlaff et al., 2012; Gallistel and Balsam, 2014), and most distinctive yet: complex properties as subjective experiences, emotions, consciousness (Cleeremans, 2011; Dehaene et al., 2014; Tononi et al., 2016) and biased behavior (Ellsberg, 1961; Gilovich et al., 2002; Moore, 2002; Machina, 2009) emerge from the brain. These emergent properties do not have any obvious correlation with higher or lower computational capability. For example, the cerebellum has more neurons than any other part of the brain, but it does not play any important role in conscious perception (Tononi and Koch, 2015).

Related to this notion, a common assumption in cognitive science is to consider the processing of information as a synonym of computation; however, it is necessary to differentiate both concepts. For instance, if the information is considered as the content of a message, this content would need a physical system to be propagated and stored. Thus, information may be understood or at least associated with a physical entity (Landauer, 1999). According to a general view, information processing can be any physical process which transforms an input into an output. Information processing can also be defined in terms of causality between inputs and outputs. Additionally, computation is mainly understood as syntactic and symbolic manipulation of information (Searle, 1990). In this sense, computation is an algorithmic and deterministic type of information processing. Although it is possible to appeal to a non-deterministic computation, in general, this non-deterministic computation can be reduced to deterministic types of simple computation at the level of a Turing machine. The problem is that brains are not just doing computation, they are also able to give interpretations and meaning to their own high-level information processing. Arguments in favor of this idea are stated from philosophical view in Searle (1990) and psychological/biological view in Cleeremans (2011).

One interesting case of computation is artificial neural networks, which could be interpreted as semi-deterministic information processing systems. Artificial neural networks evolve in a non-deterministic way thanks to self-learning and training from some given rules, which are not always explicitly programmed. These systems are semi-deterministic in the sense that it is not always possible to ensure what the net is learning, nor control the dynamic evolution of its learning process, even if deterministic learning rules have been given. Of course, it is in part because of the noise or randomness of the training data set, and/or due to predominant statistical features of the data set that were not well controlled. However, even if all these properties are controlled, it is never known what the network has learned until it is tested and even after testing; it is never possible to be sure about which node or layer encodes one or another statistical property of the data. Actually, it looks more like a domain-global and distributed characteristic than local (Christian et al., 2014). Therefore, it is not possible to fully determine or predict classically speaking the way how the net will behave. Neural and artificial neural nets are neither completely indeterminate nor determinate, but semi-determinate. Since artificial neural networks, as for example Hopfield networks (Hopfield, 1982), are inspired by biological principles (Hebb, 1949; Gerstner et al., 2012), which are in turn inspired by biological observations (Caporale and Dan, 2008), one option to introduce the semantic and meaning to artificial networks would be the implementation of interactions between subsystems as observers of each other in a context of artificial neural networks. This will be discussed in section five. Through this way, intelligence would not be only associated with deterministic logical computation but with the interaction between deterministic, semi-deterministic, non-deterministic, and perhaps quantum computation/simulations, or even new frameworks of processing of information.

While some computer and cognitive scientists might not agree with this interpretation of information and computation, it is still admissible to have processing of information without computation and intelligence without a deterministic way of processing of information. Actually, the brain apparently does it. In fact, the most important features of the brain are the result of unpredictable, nonlinear interactions among billions of cells (Ronald and Nicolelis, 2015; Haladjian and Montemayor, 2016). Science does not know the real “language” of the brain; does not know how cognitive abilities emerge from physical brains, and even more complicated, it is not certain that we have a deterministic way to explain how this emergence works.

At this point, the usual idea of digital computation in cognitive science and neuroscience should change in favor of a perspective of computation and information processing by analogy with physical systems where inputs, rules and outputs can be interpreted in a physical and global way.

The brain should not be thought as a digital computer neither in the “software” (Searle, 1990; Chalmers, 1995) nor in the “hardware” (Llinas et al., 1998; Bullock et al., 2005; Epstein, 2016). One reason is that this analogy obscures the complex physical properties of the brain. On the one hand, neuroscience and cognitive science use indiscriminately concepts as information, computation and processing of information without understanding their physical counterpart, sometimes based on the assumption of non-hardware dependency of these concepts, other times because of the assumption that the brain encodes and decodes information (and how it does so). The most common assumption is to think that activation or spikes in neurons are the only informative state. While other cells, for example astrocytes (Alvarez-maubecin et al., 2000), and non-classical integration such as neuromodulatory substances (Nusbaum et al., 2001), back-propagation (Stuart et al., 1993), among others (Bullock et al., 2005) are ignored. In addition, inactivation and deactivation states could also carry valuable information about dynamical brain states at macro and micro scale. Neurons are never in a static state and their membranes are presenting fluctuations that could still be informative (for instance, Sub-threshold oscillations). The distinctive physical brain properties and their dynamical interactions are apparently more important than in digital interpretations, what implies that hardware cannot be ignored at all. According to this point, the analogy between a drum and the brain would be more relevant than the analogy brain-computer. Drums can respond with different and complex vibration states when they are stimulated, and they can be also understood on computational terms: input (hits), rules (physical laws, physical constraints such as material, tension, etc.), and outputs (vibration, sounds, normal modes). Indeed, the brain has many more similarities with a dynamical system as a drum than with digital computers, which are based on discrete states. Drums, as well as brains, are dynamical systems with emergent and sub-emergent properties, drums have different modes of vibration, superposition, physical memory, sparse “storage” of this memory, among others features. In abstract terms, drums are also “computing” and processing information, but this information processing is a dynamical reaction from external/internal stimuli more than a formal calculation process (computation as defined above).

On another hand, computer science is missing valuable information on the attempt of replicating brain capabilities. One example is alpha, gamma or oscillations of brains in general (Buzsáki and Draguhn, 2004), synchrony (Varela et al., 2001; Uhlhaas et al., 2010), harmonic waves (Atasoy et al., 2016), among other processes which are not seriously considered in artificial intelligence, not even using artificial neural networks. Sub-emergent properties in the brain may be also important, such as plasticity changes due to the intentional practice of meditation (Lutz et al., 2004; Brefczynski-Lewis et al., 2007). These characteristics should be understood and incorporated in order to implement the social behavior in new generations of computers, machines and robots. Considering that some of these behaviors are intrinsic to biological organisms, perhaps these behaviors are not reproducible without some intrinsic constituents of information processing of biological organisms (Chappell and Sloman, 2007; Sloman, 2007) as for example oscillations or neurotransmitters.

Finally, abstractions and general concepts are really useful in theoretical terms; however, concepts as computation, information, and information processing in the brain do not have evident interpretation. Realizing that these concepts should not be used as an analogy with computers is the only way to lead us to the correct direction: Focusing on differences between brains and computers, and trying to fill the gaps without assumptions. Maybe, for many computer scientists, these comments are trivial, but what computation means for computer science is not the same as for biological science, leading to misunderstandings and misconceptions, while also the knowledge that computer sciences have about “codification” in the brain is very limited, leading to erroneous assumptions.

To sum up, sections two and three have identified some usual presumptions: (i) The assumption of a set of distinctive properties defining human being without focus on the distinctive properties of human being, (ii) intelligence related only to logical and rational thinking, (iii) brains working by analogy with hardware-independent computers, (iv) computation as synonym of information processing, and (v) brain information only “encoded” in the activation states of neurons. When differences between concepts appear, it becomes necessary to clarify some of them. That is why a subset of the features of human beings has been identified and some concepts clarified. For example, a better understanding, and definition of information processing in the context of human intelligence, where computation will be a kind of information processing among many other types, including the characteristic one to biological organisms (Chappell and Sloman, 2007). Probably, new concepts and foundations of information will be also needed, especially to understand the real language of brain cells, as a crucial theoretical starting point. These foundations should be inherent to minimal constitutive parts of physical theories and as it mentioned above, important hardware requirements, emergent, plasticity and sub-emergent properties should be considered in any attempt to replicate brains features. Thus, a computer-brain metaphor is not useful anymore, at least in the current sense. Nevertheless, it could still be possible to replicate some brains abilities thanks to new formulations of information processing and theoretical frameworks.

## Consciousness as requirement for human intelligence

Intelligence should also be considered as a whole. Intelligence is often understood as the ability to solve problems in an efficient way, thanks to other mechanisms like learning and memory. It means the maximization of the positive results in a certain solution while minimizing the negative impacts, for instance, waste of time. To do that, other processes, such as learning and memory, are also needed and associated with the definition of intelligence. In a general sense, learning has been understood as the process to gain new knowledge or improve some behavior, while the memory is the storage of this knowledge. To solve problems efficiently, it is necessary to access a certain memory that was acquired thanks to a specific learning that will modify again the memory of the system. The more intelligent is the system, the more it learns. However, in that framework, it is forgotten that emotions, subjective experiences, and cognition are deeply connected with human intelligence (Haladjian and Montemayor, 2016). They play a crucial role in learning, in the consolidation of memories, in retrieved memory and human cognition in general (Cleeremans, 2011).

Therefore, as it was stated in section A Sub Set of Human Capabilities, intelligence is better defined as the capability of any system to take advantage of their environment to achieve a goal. Specifically, human intelligence would be the ability to take advantage of their environment to keep autonomy and reproduction thanks to a balance between rational and emotional information processing. With this last definition, both main features on human thinking, reason and emotion, are merged in one global concept, together with two other features, autonomy and reproduction, that also define, altogether, the potential set of human being properties. In this context, perception, cognition, learning, and memory are key features of human intelligence considered as a whole and emerged from specific soft properties of brains, such as for example neural plasticity and oscillations. Learning and memory are intrinsically dynamic processes in the brain, changing all the time and conditional to these soft neural properties, while for computers, memory is a very static feature, mainly grounded on symbolic discretization, and in the best case, learning is driven for efficient algorithms which are also statics. Biologically, the more intelligent the system, the more balance the system has between different inner processes to achieve specific or general goals. For example, a computer is designed to make faster calculus, algorithms, and other kinds of very useful tasks, however, the computer cannot take advantage of anything that it does, in conclusion, computers are not really intelligent. Nevertheless, the last version of AlphaGo zero (Silver et al., 2017) can learn by itself and take advantage from the knowledge given as input, to improve its own performance in a specific task, as for example playing Go. Using the intelligence definition stated here, this system is more intelligent than a simple computer. By analogy, if a lizard is compared with a mouse, the later has a larger repertoire of actions, taking more advantage of their environment, than the lizard. In this sense, mice are more intelligent than lizards. It is possible to continue and even define which humans will be “more intelligent” than others looking at how they take advantage of the environment in a way that they balance both rational and emotional costs. For instance, a person who wins a discussion with his partner at the expense of their relationship is less intelligent than who wins the discussion and keep a good relationship. The crucial point is that emotions are playing an important role in classical processes of natural intelligence such as learning and memory, but they are also playing a crucial role increasing the repertoire of actions and possibilities to achieve biological goals. These new behaviors are not, paradoxically, always efficient, in a logical way, but they are the best way to achieve the goal according to the system strategy (learned by experience) even when they can interfere with rational/optimal solutions. Emotions are not just used to improve memory or learning curves; they are also useful to increase the variability and unpredictability of behavior.

Furthermore, one requirement for emotional and logical/rational intelligence, as starting point to show some of the subset human features mentioned above, seems to be what is called subjective experience (Barron and Klein, 2016) or in a more complex order: Consciousness. On the one hand, high level processes needed for moral thinking such as self-reflection, sense of confidence, error detection, understanding context, among others (Figure 1B) are essential part of consciousness and subjective experience as a whole (Gehring et al., 1993; Smith, 2009; Fleming et al., 2012). Self- reflection and sense of confidence are understood as the ability to report a mistake, like error detection, and grade the confidence of some decisions or action, even before receiving any feedback about the mistake. In fact, some researchers have suggested the intrinsic relation between social complexity associated with these processes and the emergence of consciousness (Arsiwalla et al., 2017). On another hand, humans first need to be conscious to take some complex rational decisions, to plan, and to have the intention to do something (Baars, 2005; Tononi and Koch, 2008). For example, vegetative patients and minimally conscious patients do not present signals neither planning nor having intentions to do minimal tasks (Gosseries et al., 2014), even when they could present minimal signs of consciousness (Owen et al., 2006). Planning and intentions apparently emerge when minimal signs of consciousness exceed a threshold. In fact, these minimal signs can be interpreted as predictors of recovering in minimally conscious patients (Bekinschtein et al., 2009; Casali et al., 2013). Other works are re-defining the idea of subjective experience until its minimal constitutive part and argue the existence of basic subjective experience even in insects (Barron and Klein, 2016). It would mean that complex decisions, planning, and have intentions which are needed to moral thoughts are different from consciousness, although they are closely related: Subjective and conscious perceptions are apparently previous to rational intelligence, planning, moral thoughts, and even efficient behaviors. For example, experiments in the psychology of judgment and behavioral economics have also shown that subjects tend to perform some tasks in a biased manner even if they have been trained, suggesting that logical and rational intelligence appear only after more elaborated information processing (Gilovich et al., 2002; Kahneman, 2003). It is clear that how biology implements high-level intelligence is completely different from how computer science implements it (Moravec, 1988). The whole set of human intelligence, as the capacity to take advantage of the environment, would only emerge after awareness.

The need to incorporate subjective experience and eventually consciousness to reach complex intelligence implies a complex problem which involves many different processes as awareness, emotions, subjectivity, intentionality, and attention, among others. Consciousness should be composed by all of these processes like a differentiated and unified whole, but it is not any of them. For example, it could be necessary to be aware to have emotions and subjective experiences, or maybe vice versa, and we will need them to show intentionality, attention and high-level cognitive abilities. It is also necessary to insist and distinguish that these are different processes, for instance, awareness and attention; while it is important understanding all of them as constituent parts of what we describe as consciousness. For example, at least two main processes have been identified with consciousness: (1) the fact of knowing something or what here will be understood as awareness, i.e., to become aware of something and/or perceive something internally or externally, and (2) to know that I know or do not know something, or more precisely the notion of self-conscious systems (Varela, 1975) as a “monitoring” process of this awareness and connected with the more general concept of self-reference (Varela, 1975; Kauffman and Varela, 1980; Kauffman, 1987). It is worth differentiating self-reference, as an autonomous process (where a third system emerge from its own interactions; Goguen and Varela, 1979), from other interpretations, as for instance self-monitoring as control process (where a second or third system, at the same “complex” level than others, is needed to control; Dehaene et al., 2017). Here, the notion refers to the idea of self-reference for living machines. Thus, awareness is also understood as conscious or non-conscious “contents” and self-reference is connected with conscious or non-conscious manipulations (processing) of “contents” (Shea and Frith, 2016), or what will be more precisely called “neural objects.” In this sense, subjectivity and conscious perception apparently needed to reach rational, emotional, and moral thoughts are associated with awareness and self-reference as crucial ingredients of consciousness. Nevertheless, consciousness is not reduced to the possible relationship between awareness and self-reference, it is the whole process of processes interconnected with awareness, self-reference, subjectivity, rational and emotional thoughts, among many others. Consciousness emerges from all of them as a whole (Varela and Goguen, 1978). Hence, after consciousness emerges from the interaction between these processes, human intelligence would appear as the group of strategies to take advantage of the environment thanks to the balance of emotional and rational information processing.

**Figure 2:**
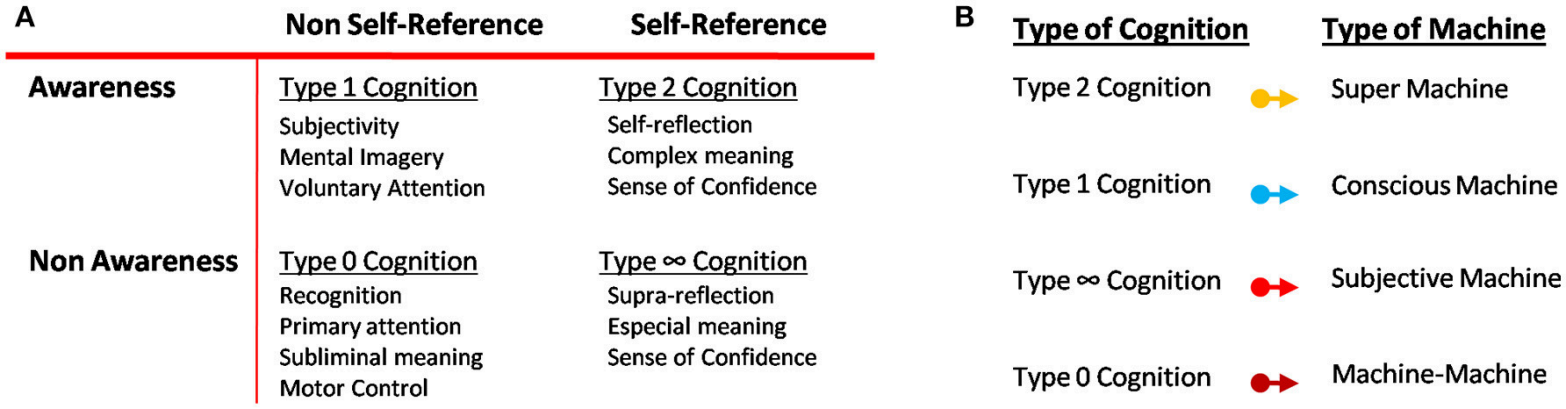
Types of Cognition and Types of Machines. **(A)** Emergent processes related to consciousness and Types of cognition defined from their relations. It is important to highlight that processes associated with moral thought are present in type 1 and type 2 cognition, but not necessarily in the other two types of cognition. **(B)** Types of machines and categories according to different types of cognition, contents, and information processing stated above.

Four types of cognition and some of their associated tasks can also be defined from awareness and self-reference (Shea and Frith, 2016; Signorelli, 2017; Figure 2A): (1) Type 0 Cognition corresponds to systems which have neither awareness of their internal or external contents nor self-reference of their internal processes. One example in humans is motor control. Motor control is the automatic control that the neural central system has to move some joints and muscles without any necessity of voluntary control or awareness. Many apparently high-level tasks in human can be classified in this category, as for example the extraction of individual word meaning and primary attention sometimes called priming. (2) Type 1 Cognition is defined as the type of cognition emerged when a system is aware of their contents. In other words, it is aware of the elements that the system needs to manipulate and solve particular or general problems, but the system does not monitor this manipulation. It can be also associated with a holistic kind of information. For example, when subjects answer very quickly to some apparently intuitive questions but their answers are normally wrong (Fallacy questions). Type 1 cognition also involves mental imagery, emotions, voluntary attention and most of our subjective capabilities as to be aware of the experience of color or pain, among others. (3) Type 2 Cognition appears when the system is aware of their contents and also has self-reference capability as the ability to manipulate them. This type of cognition involves the high-level cognitive capabilities defined above and needed for human morality. Some tasks, which are part of this type of cognition, can be: the ability of self-reflection; rational thinking; detection of error even before receive any clue about the mistake; sense of confidence, before and after any decision; complex meanings; voluntary and quick learning, among other interesting features of human thinking. (4) Finally, Type ∞ cognition incorporates the manipulation of contents without awareness of their contents. In other words, the system has self-reference, but it cannot extract meaning either from their manipulation nor their contents. It could be like an automaton, and actually, there is not a biological example of this category.

These categories will help us to classify the kind of machine and the characteristics needed as a requirement to reach or overcome human cognitive capabilities. These ideas may imply that to reproduce high-level of human intelligence following biological principles, it is necessary but not sufficient to introduce first, subjective and conscious behavior in machines at early stages to reach the type 1 and type 2 cognition of human beings. Then, the question of overcoming humans is intrinsically related to the question of build conscious machines. In this way, machines will be classified by analogy to the cognitive level that can reach according to the types of cognition emerged from awareness and self-reference (Figure 2B). These two processes would be previous to complex kind of cognition, as for example type 2 cognition, voluntary learning and complex memories, but only sufficient features to overcome humans if autonomy, reproduction, and morality are also reached. In other words, the only way to reach human brains would be making conscious machines capable of reproducing emotional human intelligence, in addition to logical intelligence, and keeping their autonomy, reproduction capacity, and reaching moral/ethical thinking. Otherwise, machines will never surpass humans.

Therefore, in order to implement high-level-computers, that is to say, computers-like-brain, it will be necessary to focus on conscious human capabilities, and how they are impacting the information processing of the system.

## Dynamic of consciousness

Any understanding of consciousness should try to explain a huge set of behaviors associated with consciousness. Chalmers defined some of them (Chalmers, 2013), ranging from apparently “simple” tasks (called third-person data) such as perceptual discrimination of stimuli, integration of different sensory modalities, automatic and voluntary actions, accesses and reportability of internal states, differences between sleep and wakefulness, to phenomena even more difficult to explain (called first-person data), for example perceptual experiences (e.g., the experience of color), bodily experiences (e.g., pain and hunger), mental imagery, emotional experiences, among others. Some useful distinctions to study consciousness also point out the differences between studies of wakefulness and studies of conscious perception or awareness (Chalmers, 2013). The first mechanism would describe the differences between, for example, sleep, vegetative and awake conditions, while the second one tries to explain when and how a perception become consciously perceived, in other words, when we become aware of something (Dehaene et al., 2014). In the end, it is expected that both approaches will help to answer important questions about mechanisms of consciousness, however, these studies do not always include subjective experience, which is assumed to be solved after the understanding of the mechanisms of wakefulness and awareness.

For example, one intriguing characteristic observed from the comparison of subjects in awake condition vs. sleep, vegetative and anesthesia condition is that the neural activity driven by an external stimulation spreads through different areas of the brain when subjects are awake, but remains local when they do not (Rosanova et al., 2012; Casali et al., 2013; Sarasso et al., 2014). Experiments with transcranial magnetic stimulation (TMS) and electroencephalogram recording (EEG) demonstrated this effect. For awake condition, pulses driven by TMS generate richer and sequential EEG signals in different brain areas, and remarkably, the peak of these global activities is lower than in other conditions, where awareness is absent. This signal has been linked with the integration of the brain activity but it is still not clear how integration takes place, which mechanisms allow the global diffusion of each pulse, and why in other than awake condition, the integration remains local.

Additionally, consciousness, awareness and conscious perception, apparently, are not matter of capacity of computation. The brain should not be considered as a computer, neither doing any computation like a computer, as stated above. Although, if someone would like to insist, the brain capacity can be roughly estimated around 20 petaFLOPS, assuming 100 billions of brain cells, 200 firings per second, and 1,000 connections per cell [see other approximations (Martins et al., 2012)], whereas independently of any approximation, 80% of these brain cells (hence its computational capacity) are in the cerebellum, which does not play any important role in conscious perception (Tononi and Koch, 2015). By comparison, the most powerful computer has 93 petaFLOPS [Sunway TaihuLight (Dongarra, 2016; Fu et al., 2016)]. It is however really unlikely that someone ensures that this computer is aware despite its bigger computational capacity. AlphaGo is another example that computational capacity is not the key to improve or reach high-level tasks. The last version AlphaGo zero defeats previous AlphaGo versions but uses less computational resources, suggesting the importance of learning algorithms and neural network architecture to solve complex high-level tasks (Silver et al., 2017).

**Figure 3:**
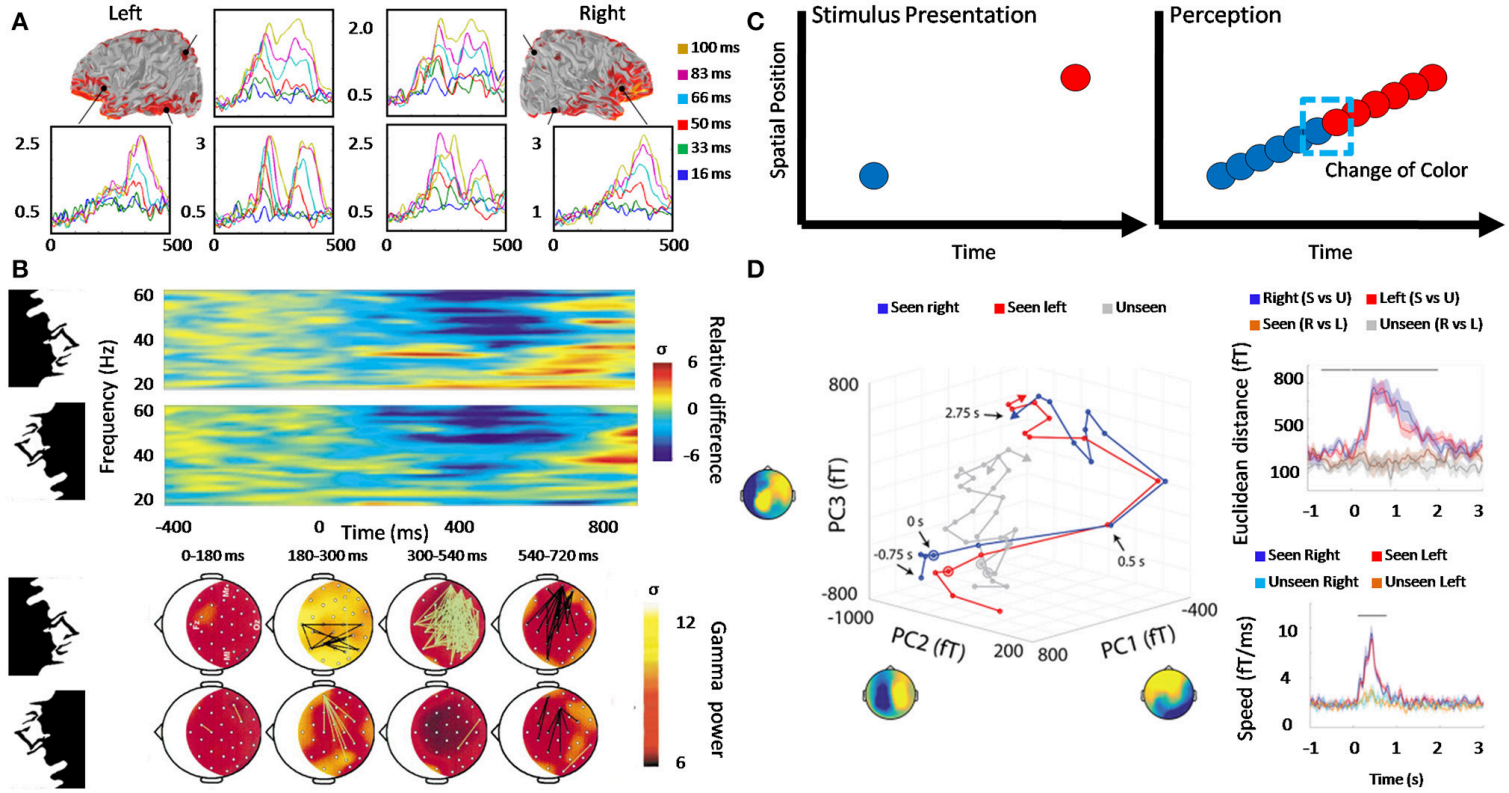
Neural dynamic associated with awareness and some experimental evidence. **(A)** Three cortical areas recorded in left and right hemisphere (posterior parietal, posterior ventral temporal, and inferior frontal) present slightly different types of activity evoked by masked targets. The peak in the condition of maximal visibility is associated with P3b (around 370 ms). Two phases of cortical activation can be recognized, the first previous to 300 ms corresponds to the activity from the occipital pole toward both parietal and ventral temporal sites. The second phase, after 300 ms, is characterized by a high-amplitude activity, which mainly appears in ventral prefrontal cortex together with a re-activation of all previous posterior areas. Colors represent six different conditions where the time of the target-mask stimulus onset asynchrony increased in value, allowing the same stimulus to cross a hypothetical threshold from subliminal processing to conscious perception. Adapted from Del Cul et al. (2007). **(B)** When high contrast faces are presented to normal subjects a long distance synchrony during face-recognition appears around 200 ms at 40 Hz frequency band. Additionally, the effect disappears if the same stimulus is reversed, avoiding the recognition. Another period of synchrony also appears during the motor response and crucially, a transient phase scattering between both synchronic phases showed a decrease in the probability of synchrony. Upper chart is the time-frequency synchrony activity and inferior chart corresponds to the perception condition mapped onto surface electrodes, where black lines indicate a significant level of synchrony, and green lines indicate a marked phase scattering between electrodes. Adapted from Varela et al. (2001) and Rodriguez et al. (1999) with permission of Springer Nature. **(C)** In the color phi phenomenon, two disks are shown at different positions with a rapid succession, inducing the illusion of only one disk which changes the color around the middle trajectory. This phenomenon is contrary to a continuous perceptual dynamic because the observer does not have the opportunity to know in advance the new disk color, especially if the perception is not retrospectively built. Adapted from Herzog et al. (2016). **(D)** Activity trajectories in Principal Component (PC) space of visual conscious perception (red and blue) are different than unconscious perception trajectories (gray). For simplicity, only the first three PCs for subject 2 are shown. The upper-right chart shows the group average Euclidean distance between temporal points for each trajectory [blue right (seen vs. unseen), red (left seen vs. unseen), purple seen (right vs. left), and gray unseen (right vs. left)]. Inferior-right chart corresponds to group average speed of activities trajectories at each time point. Horizontal black lines indicate significant difference (2-way ANOVA, *p* < 0.05, cluster based on permutation test). Adapted from Baria et al. (2017).

Nevertheless, evidence has shown that conscious perception needs between 200 to 400 ms (Dehaene and Changeux, 2011) while the processing and integration of information at low-level tasks only need 40 ms. In other words, when we consciously perceive, any processing of information is temporally decreasing between 500 up 1,000%. Experiments, where subjects were exposed to masked stimuli (words or pictures which are masked by previous stimuli), have showed that conscious perception (i.e., subjects report seeing the stimulus) is correlated with a positive peak in Event-related potentials (ERPs) which appear 300–500 ms after the stimulus presentation (Figure 3A; Dehaene and Changeux, 2011; Herzog et al., 2016). It is interesting to notice that the neural activity for some cortical regions seems to show a shortly decrease of activity, while other areas showed a later peak around 300–400 ms (Del Cul et al., 2007). This response is called P3b and has not uniquely associated with perception but also with attention and memory processes. The mechanism suggested as an explanation of P3b is a sustained stable activity in recurrent cortical loops. Another mechanism proposed as a marker of conscious perception, called synchrony, has been also observed within a window of 200–400 ms. High-contrast human faces were presented in normal and inverted orientation (Rodriguez et al., 1999), and synchrony was observed around 250 ms each time that faces were recognized. Synchrony was mainly between occipital, parietal and frontal areas (Figure 3B). Furthermore, a new pattern of synchrony (in the gamma range) emerged around 720 ms during the motor response. One notable phenomenon from this experiment is the phase scattering presented between these two synchronic responses (Varela et al., 2001). At this time, the probability of finding synchrony between two EEG electrodes was below the level observed before stimulation (Figure 3B). This phase scattering and phase synchronization show an interesting kind of alternation or maybe interference, which should be explained by any theory of consciousness.

A recent experiment has additionally demonstrated a transient neural dynamic during visual conscious perception (Baria et al., 2017), challenging sustained activity mechanisms as broadcasting and integration, and suggesting initial-state-dependent neural dynamics. Neural activity, previous, during and post stimuli, was measured with magnetoencephalography (MEG). Subjects were asked to recognize the direction of Gabor stimulus (left or right) and inform if the stimulus had been consciously perceived (stimuli were manipulated to induce around 50% of conscious perception in each subject). Then, neural activity was divided into different frequency bands to calculate the multi-dimensional state space trajectory computed with principal component analysis (PCA). In the band 0.05–5 Hz, trajectories of conscious (seen) and unconscious (unseen) trials were clearly separable (Figure 3D) by Euclidean distance (Figure 3D upper right). Crucially, the speed of population activity, measured as a point trajectory in the state space vs. time (ms), showed an acceleration and switch in dynamics after stimulus onset, with a peak around 400 ms (Figure 3D inferior right). Moreover, conscious stimuli perception was predicted from the activity up to 1 second before stimulus onset (Baria et al., 2017).

Until now, it is not clear that integration, P3b response and/or synchrony are markers of conscious perception or awareness (Gaillard et al., 2009; Mudrik et al., 2014; Silverstein et al., 2015) and there is no consensus if one exclusive marker can be actually identified. Even so, they can still be markers of “contents” construction at conscious and unconscious level. Most theories about consciousness assume that the construction of contents of consciousness is part of the same phenomenon that they call consciousness, in the sense of awareness. Nevertheless, it is equally reasonable to think that the constructions of contents and awareness are two different dynamics of one process, as transient dynamics suggest, or even two completely different processes. One alternative is to think that the construction of contents is a separated process and previous to the process of becoming aware of these contents. So, we should speak about neural objects, also avoiding “the container” interpretation of consciousness. If this is correct, much recent research on consciousness and conscious perception would be inferring information about the construction of these neural objects that are not necessarily associated in a causal way with consciousness itself. Thus, awareness is one process to explain, and the construction of a perception or objects of consciousness would be another. Integration, P3b and synchrony would be, in this sense, part of the construction of neural objects, but not part of the awareness moment where the object becomes part of our conscious perception. Chronologically, one first stage of information processing should be the constructions of these objects and a second stage would be the awareness of them. These processes would be independent and only from their interactions, as the observer and the observed at the same time, the conscious perception of internal and external neural objects would emerge avoiding the “Cartesian theater” interpretation (Lycan and Dennett, 1993). In other words, it is admissible to be aware without conscious perception of some objects, and “perceive” without awareness about this perception.

**Figure 4:**
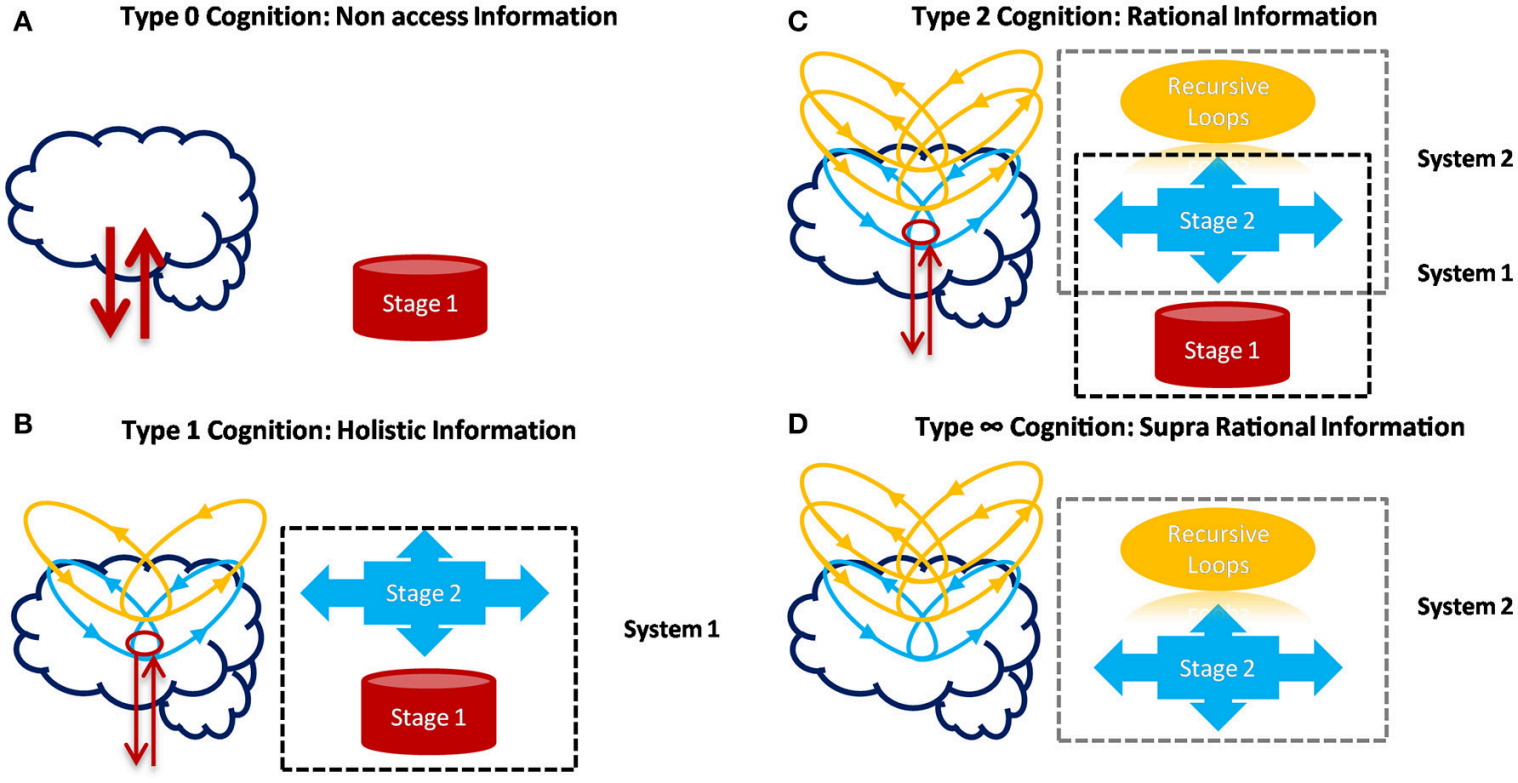
Types of Cognition, their relation with possible systems and stages of information processing. **(A)** Stage 1 corresponds to automatic and non-conscious processes (classical information) in principal layers. It is associated with Type 0 Cognition. **(B)** Stage 2 is related to awareness and conscious perception as holistic information (Type 1 Cognition) when two or more principal layer interact. Both stages form the non-classical system 1 (linked with psychological features), which is not necessarily deterministic in a classical way. **(C)** Recursive loops of stage 2 would correspond to conscious manipulation processes of contents (Self-reference). From the interaction of stage 2, their recursive loops and re-entry of information with system 1, another classical and deterministic system 2 would emerge. **(D)** The system 2 alone and without interacting with system 1 would correspond to Type ∞ cognition. This type of cognition is a hypothetical/speculative scenario emerged by the relations of Awareness and Self-Reference components in our theoretical framework. However, its existence is doubtful considering that system 2 in living beings, would need system 1 to emerge.

Additionally, conscious perception is not always differentiated in awareness and self-reference, but here the distinction is made in order to define clearly different levels of cognition, which would describe two processes of the same conscious phenomenon. In other words, it is possible to state that information processing can be divided into different stages (Figure 4), where awareness is related to one of these stages and self-reference with the recursive processing of this stage. The differences between fast time processing for cognition type 0 (~40 ms) and a slow time processing for type 1 (~200 ms) have stimulated the idea of Two-Stage Model (Herzog et al., 2016). This is to say that the flux of activity (or inactivity) would need at least two different stages (from which types of cognition emerge), where the first stage corresponds to automatic, non-voluntary control and unconscious information processing, while the second stage would involve a break in this dynamic to allow awareness. Furthermore, it is proposed here that the recursive processing of awareness within the same neural objects will allow the emergence of self-reference process (Figure 4).

Other experiments also suggest a discrete mechanism instead of a continuous perception mechanism (VanRullen and Koch, 2003; Chakravarthi and VanRullen, 2012; Herzog et al., 2016). For example, evidence for the discrete mechanism of perception comes from psychophysical experiments where two different stimuli are presented with a short time window between each other. In these experiments, subjects perceived both stimuli as occurring simultaneously, suggesting a discrete temporal window of perception integration (VanRullen and Koch, 2003; Herzog et al., 2016). The most relevant experiment supporting a discrete perception is the color phi phenomenon (Figure 3C). In two different locations, two disks of different color are presented in a rapid succession. The observer perceives one disk moving between both positions and changing the color in the middle of the trajectory. Theoretically, the experience of changing color should not be possible before the second disk is seen. Therefore, the perception should be formed retrospectively, which is contrary to continuous theories (Koler and VonGrünau, 1976; Bachmann et al., 2004; Herzog et al., 2016).

Another characteristic is the apparent “interference” between different types of information processed in human conscious behavior. For instance, rational calculations (e.g., resolve a mathematical problem) interfere with kinaesthetic performance (Shea and Frith, 2016). To illustrate, solving a mathematical equation while cycling or dancing at the same time can be practically impossible. This observation suggests that conscious perception would be imposing a balance between different processes. Computational interpretation of this observation will try to explain the interference between different kinds of information as a competition for computational capacity or resources. However, as it is stated above, computational capacity apparently is not playing any crucial role in perception. This analogy also assumes processing of information in a digital way, which could not be the best approach to understand the brain.

Finally, some results from behavioral economics and decision making have shown that cognitive biases are not according to classical probability frameworks (Pothos and Busemeyer, 2013). It means that it is not always possible to describe emergent brain properties with classical and efficient probabilities way. For example, when one tries to explain, for one side, the biological mechanisms in the brain, and on the other, the human psychological behavioral, crucial differences appear. Some research and theories have shown that the dynamics of neural systems can be interpreted in a classic probabilities framework (Pouget et al., 2000; Quiroga and Panzeri, 2009), like good estimator and predictor of external stimuli. While other results, mainly from economic psychology, show cognitive fallacies (Ellsberg, 1961; Gilovich et al., 2002; Moore, 2002; Machina, 2009). These results are incompatible with the classical probability theories (Pothos and Busemeyer, 2013) and can be reconciled only after an extra processing of information in experimental subjects. Therefore, these disconnections between some neural activities in the brain (as classical systems), the emerged human behavior and some of their cognitive capabilities (non-classical systems), and then another possible classical system suggest complex multiple separate systems with interconnected activity (Figure 4C). How can some cognitive capabilities, with apparently non-classical dynamic, emerge from apparently classical, or semi-classical systems as neural networks? It is one open question that any theory of consciousness should also try to explain.

## An alternative: consciousness interaction hypotheses

If consciousness is not a matter of computation capacity, given that temporal efficiency decreases in its presence, it could be due to its architecture. Many theories have tried to explain how consciousness emerges from the brain (Dehaene et al., 2014; Tononi et al., 2016). However, these theories are incomplete although they might be partially correct. The incompleteness is in part because most of these theories are descriptions of the phenomenon, instead of explanatory theories of the phenomenon. By way of example, Classical Mechanics and Theory of evolution are explanatory theories (although an explanatory and/or complete theory does not ensure that it is correct). Descriptive theories focus on how the phenomenon works, use descriptions without causal mechanisms even when they claim it, and without deductive general principles, i.e., they often start from the object of study to deduce specific/particular principles rather than deducing general principles and in consequence explaining the object of study. Furthermore, incomplete theories do not answer one of these fundamental questions: What is “the object of study”? How does it work? Why? Most commonly, they do not explain “why” something works as it works. In other words, these theories may partially explain and/or describe how consciousness emerges, but they do not explain and do not solve the entire problem. The problem, according to Chalmers (1995, 2013) is to explain both the first-person data related to subjective experience and the third-person data associated with brain processes and behavior. Most of the modern theories of consciousness focus on the third-person data and brain correlates of consciousness without any insight about the subjective experience. Moreover, some of the questions stated above as for example the phase scattering, the transient dynamics, the decrease in the peak of EEG activity driven by TMS, the two stages and two systems division, are not explained, and actually, they are not even well-defined questions that theories of consciousness should explain. Finally, these approaches try to explain awareness and conscious perception in a way that is not clearly replicable or implementable in any sense, neither with biological elements. Some theories also use the implicit idea of computability to explain, for example, conscious contents as the access to certain space of integration; and competition for space of computation in this space, to explain how some processes lose processing capacity when we are conscious.

**Figure 5:**
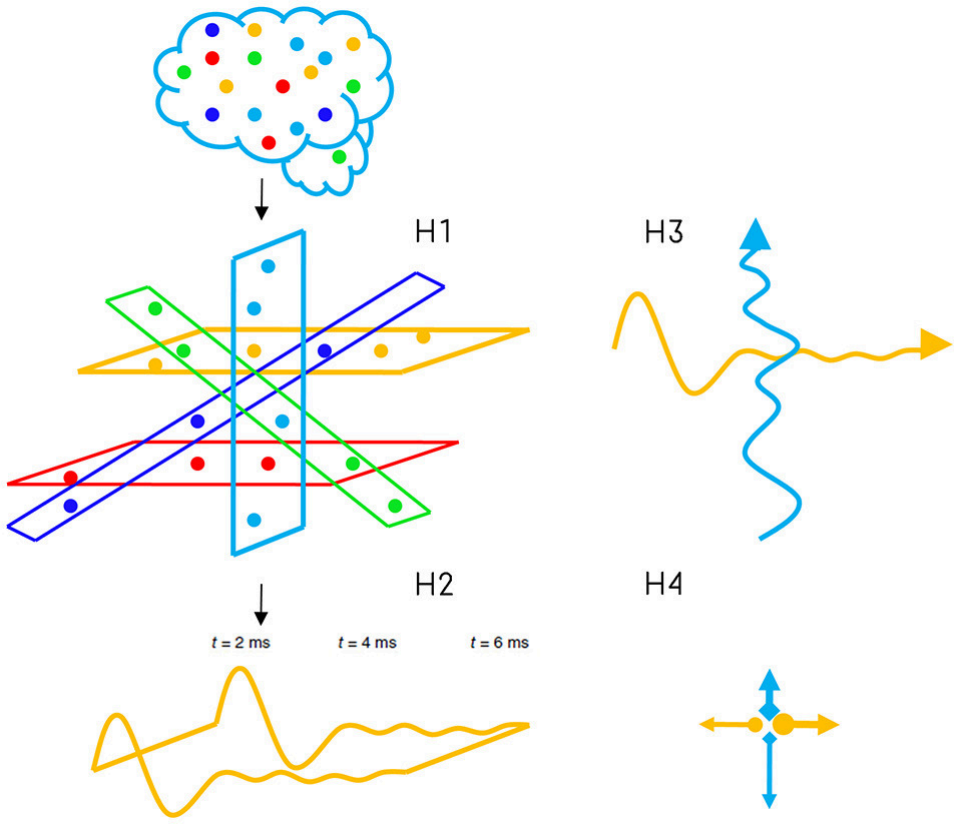
Consciousness Interaction Approach and its four hypotheses. Hypothesis 1: the architecture of the brain can be divided into different independent processing layers (network of networks); hypothesis 2: each layer independently processes information to solve each particular problem; hypothesis 3: two layers activated at the same time can interact/interfere with optimal information processing of each other at different levels, and; hypothesis 4 suggests that this interaction/interference/superposition between at least two principal/independent layers (for example horizontal and vertical) would be associated with the general mechanism of consciousness.

Another complementary alternative is to understand consciousness as intrinsic property due to the particular form of information processing in the brain. Here, consciousness will be interpreted in this way, as the dynamic interaction/interference (which can be superposition or interference) of different neural networks dynamics, trying to integrate information to solve each particular network problem. More specifically, the brain could be divided into different “principal layers” (topologically speaking, it corresponds to the architecture component) which are also composed by different levels of layers (hypothesis 1), each principal layer as one kind of neural network interconnected at different levels with other networks (Figure 5). Each principal layer can process information thanks to oscillatory properties and independently of other principal layers (hypothesis 2); however, when they are activated at the same time to solve independent problems, the interaction generates a kind of interference on each intrinsic process (hypothesis 3, the processing component). From this interaction and interference would emerge consciousness as a whole (hypothesis 4). I will call it: Consciousness interaction hypotheses. Consciousness would be defined as a process of processes which mainly interferes with neural integration. These processes are an indivisible part of consciousness, and from their interaction/interference, consciousness emerges as a field of electrical, chemical, and kinaesthetic fluctuations.

There are two possible interpretations about these principal layers: the first one is the idea that these principal layers are formed by areas structurally connected, and the second possibility is that they are formed by areas only functionally or virtually connected. In the latter, the functional connectivity should be defined by phases and frequency dynamics to avoid in part the bias about neural activity mentioned above. Experiments and new analyses motivated by these ideas should solve which interpretation is the optimal one. Additionally, the nature of the interference suggested here can sometimes take the form of superposition and other times the form of subtraction in the threshold and/or sub-threshold oscillatory activity associated with neural integration, in two or more principal layers. This interference as a superposition or subtraction would be one possible mechanism to one independent neural process interferes with the other and vice versa (this is not necessarily excitatory and inhibitory neural interactions). Once this interaction has emerged, each principal layer monitors the other without any hierarchical predominance between layers, and if one process disappears, awareness also disappears. In this sense, each principal layer cares about its information processing and the other information processing which can affect them. The oscillatory activity at individual neural layers can be interpreted as one stage (classical information), and when the new activity emerges thanks to interference between principal layers, the second stage would emerge (non-classical information) forming one system. Then, the recursive action of the second stage would allow the emergence of a second system. In the end, both systems as a whole of layers and interactions would be the field of consciousness which cares about its own balance to be able to solve each layer problem.

The idea of “care about something” could also explain in part the subjectivity experience. Each layer cares about some states more than others, based on previous experiences and learning (Cleeremans, 2011), but also grounded on the intrinsic interaction between principal layers defined above, which allow them to solve their information processing problems. In other words, depending on the degree and type of interference for a certain experience, the system would feel one or another feeling, even if the external stimulation (perceptually speaking) is the same for many subjects. The subjectivity, at least preliminarily, would not directly be more or less neural activity. It would be related to the type and degree of interaction between principal layers emerged by learning, balancing processes thanks to plasticity and sub-emergent properties, which all together try to keep the balance of the whole system. This plasticity would be part of emergent and sub-emergent properties of dynamical systems, probably driven by oscillations and neurotransmitters. The system would be trained, first by reinforcement learning and later through also voluntary and conscious learning.

These hypotheses might allow us to replicate some neural activities illustrated above, some features of conscious behavior and to explain, for example, why the brain is not always an efficient machine as it is observed in cognitive fallacies, why decisions are not always optimal, especially in moral dilemmas, why it is possible to observe an apparent decrease in processing capacity between different types of information processing in human conscious behavior when we try to perform rational vs. kinaesthetic tasks. The sustained interference mechanism would break the stability in principal layers triggering different responses in each one, breaking synchrony, local integration and spreading activity and de-activity around principal layers. It could explain in part the transient dynamic, the scattering phase between two synchronic phases associated with conscious perception and motion reportability, or why the activity after TMS in awareness is globally spread, and more interesting, it would allow us to implement a mechanism on other machines than biological machines, if important soft properties and physical principles of brains, as plasticity and oscillations, are correctly implemented in artificial systems. Although these ideas still do not answer the “why” question of a complete theory of consciousness, they are part of a global framework on codification, processing of information, mathematical category and physical theories, which will intent to answer that question and will be developed in further works.

Some important differences of this framework with previous approaches are: (1) awareness would emerge from the property of breaking neural integration, synchrony and symmetry of the system; (2) conscious perception would correspond to dynamics operations between networks more than containers formed by networks in which to put contents. In this sense, consciousness is a distributed phenomenon by essence and the semantic of “neural objects” should be used instead of contents; (3) consciousness would be related to mechanism of oscillatory superposition, interference and sub-emergent properties as oscillatory plasticity; (4) consciousness interaction hypothesis could be an implementable mechanism for artificial intelligence.

Finally, one crucial observation emerges from this discussion. Consciousness interaction hypothesis requires a balance of interaction/interference between different processes involved in its emergence to keep, in fact, the interaction. Otherwise, one principal layer would dominate the interrelated activity, driving the activity in other layers without exchange of roles, which is the opposite approach (during other non-conscious conditions, for example, it could be the case). That is why extraordinary capacities in some processes are compensated with normal or sub-normal capacities in other processes of information when we are conscious.

## Types of cognition and types of machines

Consciousness interaction is a different framework, therefore it is necessary to re-interpret some definitions from previous theories about consciousness (Dehaene et al., 2014). **Conscious states** as different levels of awareness (vegetative, sleep, anesthesia, altered states, aware) would correspond to different types and degrees of interaction or interference between different networks. In this sense, coma patients would miss some crucial interactions between some principal layers which are important for “neural objects” constructions; while during anesthesia, the activity of some principal layers may be only locally affected, losing the optimal balance between layer interactions/interference. In consciousness interaction hypothesis, consciousness is not a particular state neither has possible states; this is a crucial difference regarding common definitions and theories. Consciousness should be interpreted as an operation/process itself. **Contents of consciousness** as elements or information in the external or internal world which at times are part of our conscious perception, would correspond to superposition of different oscillation on certain “intersection points” of interference between networks or the network points (nodes) which are influenced/affected by this interference/interaction (probably in a scattered/sparse way). These “neural objects” can be formed even without awareness. In this case, the neural object is restricted to the universe of one principal layer and their local dynamic. However, they become part of the conscious perception only when two or more principal layers start to share these elements to solve their layer problems. Only at this moment, a neural object appears as part of the field of consciousness. Finally, **conscious processing** is normally defined as the operations applied to these contents/neural objects. In consciousness interaction framework, it would correspond to constants or sustained “loops” of interference/interaction on this “intersection points” and its dynamic evolution (probably through sub-threshold resonant circuits).

With similar definitions (without this particular interference interpretation) and their relations, Shea and Frith have identified four categories of cognition (Shea and Frith, 2016) depending if neural objects and cognitive processes are conscious or not. In previous sections, these four types of cognition were re-defined (Figures 2, 4) from the inter-relation between awareness and self-reference. In summary, Type 0 cognition corresponds to cognitive processes which are not conscious neither in their neural objects nor operations applied to these objects. Type 1 cognition is a set of cognitive processes where neural objects are consciously perceived, however operations on them are not manipulated. Type 2 cognition would correspond to neural objects and operation on these objects consciously perceived and manipulated. Finally, what I have called Type ∞ cognition (Signorelli, 2017) can be understood as cognition without any kind of neural object consciously perceived, but operations on these objects are consciously manipulated. According to these definitions (Figures 2, 4), it is also possible to relate these categories with four categories of machines and their information processing capabilities (Signorelli, 2017): (1) The **Machine-Machine Type 0 Cognition** would correspond to machines and robots that do not show any kind of awareness. These systems cannot know that they know about something that they use to compute and solve problems. Machine-Machine is not intelligent according to the general definition in section A Sub Set of Human Capabilities and their processes are considered low cognitive capabilities in human. Examples are robots that we are making today with a high learning curve. (2) **Conscious-Machine Type 1 Cognition** would have awareness and all the processes of type 1 cognition in humans. This is a very smart machine, however, it cannot control voluntary their inner manipulations even when they can extract meanings of their own “contents.” As well as humans, they will show wrong answers to simple questions as for example cognitive fallacy questions, mainly because the system accesses to a wider range of information thanks to first levels of interference/interaction between networks (Holistic information), however, some optimal or specific algorithmic calculations may become intractable. (3) **Super Machine Type 2 Cognition** would be the closest machine to human, at least cognitively speaking. If this machine can reach awareness and self-reference in the sense illustrated here (not only computationally), they should show some kind of “thoughts” associated with consciousness as a whole of rational and emotional processes. In this case, they will have some moral thinking, even when their moral can be completely different than the human moral. The moral thinking is not necessarily restricted to the human morality, because as also happen in different human communities and even human subjects, machines may develop their own type of morality, and this morality can also be non-anthropocentric. Nevertheless, the requirement for any type of moral thinking is the attribution of correct and incorrect behaviors based on what the system cares about the environment, peers and itself, according to a balance between rational and emotional intelligence. If the machine has the ability of awareness and self-reference, they will develop, or they already developed self-reflection, sense of confidence, some kind of empathy among other processes mentioned to reach moral thoughts. In these machines, “contents” are conscious and the cognitive process is deliberate and controlled thanks to a recursive and sustained interference/interaction at certain intersection points from different networks (e.g., reasoning). (4) **Subjective-Machine Type** ∞ **Cognition** are different than humans, even if they could reach some important features of human intelligence. They are defined according to type ∞ cognition, where awareness is missed but self-reference would still be there. A clear analogy with humans is not stated here, even when the presence of self-reference as a kind of monitoring process without awareness could be reported in humans. However, the hypothesis about this type of machines is related to Supra reasoning information emerged from organization of intelligent parts of this supra system (e.g., Internet), where systems would show some special kind of self-reflection, sense of confidence, even when they will probably not be able to extract meaning of their own “contents,” or if they can, it will be especially different than humans.

Some previous works have been also tried to generalize and characterize some features of consciousness and their connection with types of machines and/or artificial systems (Aleksander and Morton, 2008; Wang, 2012). For example in Arsiwalla et al. (2017), even though that article still keeps a computational view of consciousness and social interactions, they conclude that consciousness is not only due to computational capacity and put emphasis in social interactions (which can also be related to emotions) as a trigger of consciousness. Another example is Gamez (2008), where some categories defined can be close to some types of machine mentioned above. However, some crucial differences with these articles are: (1) here, types of machines directly emerge from previous theoretical and experimental definitions of types of cognition. In this context, types of machines are general categories from the definitions of cognition and its relation with consciousness. (2) Additionally, here, it is not assumed any special optimization processes to achieve consciousness, actually quite the contrary, interference processes as non-optimal processes and some still missing properties of soft materials/brains would be associated with its emergence.

Due to these non-optimal processes, each type of machines has limitations (Signorelli, 2017, 2018). For instance, conscious machine type 1 cognition will reach consciousness but it does not have strong algorithmic calculation capabilities or rational/logical intelligence, because accuracy is lost in favor of consciousness as fast access to holistic information. Subjective machines type ∞ cognition probably will not be able to interact physically with us, and even less dance like us or feel like us, however, it is the most likely scenario where machines and computers would overtake some humans capabilities, keeping the current hardware in a non-anthropomorphic form. For this machine, the subjective experience could be something completely different to what it means for humans. In other words, Subjective Machines are free of human criteria of subjectivity. Eventually, Super Machine is the only chance for AI to reach and exceed human abilities as such. This machine would have subjective experiences like humans, at the same time that it would have the option to manipulate the accuracy of its own logic/rational process; however, it is also vulnerable to what subjective experiences imply: the impact of emotions in its performance and biased behavior as humans.

## Implications for artificial intelligence and conscious machine

Any attempt to accomplish conscious machines and try to overcome human capabilities should start with some of the definitions stated previously. First, it is necessary to define a set or subset of human capabilities which are desirable to imitate or even exceed. This is, actually, a common approach, the only difference is the kind of features which have been replicated or attempted to replicate. According to this work, most of them are still low-level cognitive tasks for brains. Also in this article, the subset can be considered a very ambitious group of characteristic: Autonomy, reproduction and moral. Autonomy is already one characteristic considered in AI. Research is currently working to obtain autonomous robots and machines, and nothing opposes to the idea that eventually an autonomous robot can be created. It would probably not be autonomous in the biological sense, but it could reach a high-level of autonomy. The same can be expected for reproduction. Machine reproduction will not be a reproduction as in biological entities, but if robots can repair themselves and even make their own replications, the reproduction issue can be considered reached, at least functionally speaking. However, it is not obvious that genuine moral thinking can be achieved by only improving computational capability or even learning algorithms, specifically, if AI does not add something which is an essential part of the human being: consciousness.

Moreover, when some characteristics of human brains are critically reviewed, consciousness is identified as an emergent property that requires at least two other emergent processes: awareness and self-reference. Thanks to these processes, among others, it is expected to develop high-level cognition which involves processes as self-reflection, mental imagery, subjectivity, sense of confidence, etc, which are needed to show moral thinking. In other words, the way to reach and overcome human features is trying to implement consciousness in robots to attain moral thinking.

However, to try to implement consciousness in robots, a theory is needed that can explain, biologically and physically speaking, consciousness in human brains, dynamics of possible correlates of consciousness, the psychological phenomenon associated with conscious behavior and at the same time, explore mechanisms which can be replicated into machines. It should not be mere descriptions of which areas of the brain are activated or which are the architectures of consciousness, if the interaction between them, from which consciousness would emerge, is not understood. Therefore, the understanding of emergent properties is not enough and the consideration of crucial plasticity properties of the soft materials in biology, as oscillations, stochasticity, and even noise are very important to also understand sub-emergent properties as plasticity changes influenced by voluntary or conscious activity. For one side, a more complete theory of consciousness is needed, which relates complex behavior with physical substrates and for another side, we need neuromorphic technologies to implement these theories.

One of the main attempts of this paper was to show a possible structure for consciousness, founded on a non-intuitive kind of interaction: oscillatory superposition and interference between networks of networks defined as structural and/or functional organizations changing dynamically. These principal networks try to solve particular problems, and when all of them are activated, sharing and interfering on their own oscillatory processes as a whole, the field of consciousness would emerge as a process of processes. Additionally, another main attempt explored here was to make evident some paradoxical consequences of trying to reach human capabilities. Thus, types of cognitions were defined not only to show different conscious processes, but also to show that from these categories, it is possible to define four types of machines regarding the implementation of consciousness into machines, and their limitations.

For example, if we can reach the gap to make conscious machine type 1 or 2 cognition, these machines will lose the meaningful characteristics of being a computer, that is to say: to solve problems with accuracy, speed and obedience. Any conscious machine is not a useful machine anymore; unless they want to collaborate with us. It means the machine can do whatever it wants; it has the power to do it and the intention to do it. It could be considered a biological new species, more than a machine or only computer. More important: according to our previous sections and empirical evidence from psychology and neuroscience (Haladjian and Montemayor, 2016; Signorelli, 2017), it is not possible to expect an algorithm to control the process of emergence of consciousness in this kind of machines, and in consequence, we would not be able to control them. In other words, even if it were possible to replicate consciousness and high-level cognition, each machine would be different to the other in a way that we are not going to control. If someone expects to have a super-efficient machine, it would be quite the contrary, each machine would be a lottery just as it is when people meet each other.

With this in mind, three paradoxes appear. The first paradox is that the only way to reach conscious machines and potentially overcome human capabilities with computers is by making machines which are not computers anymore. If it is considered that a subset of main features on machines is the capacity to be accurate and fast solving problems, from comments above, any system with subjective capabilities is not accurate anymore, because if they replicate high-level cognitions of human, it is also expected that they will replicate the experience of color or even pain, in a way that it will also interfere with rational and optimal calculations, as well as in humans. The second paradox is that when we make conscious machines type 1 and/or type 2 cognition, a process of interference, due to consciousness, will affect the global processing of information, allowing extraordinary rational or emotional abilities, but never both extraordinary capabilities at the same time or even in the same individual, due in part to how the intrinsic and non-controlled emergent processes associated with consciousness would work. In fact, if the machine is a computer-like-brain, this system will require a human-like-intelligence that apparently also requires a balance between different intelligence, as stated above. Hence, machines type 1 or type 2 cognition would never surpass human abilities, or if it does, it will have some limitations like humans. The last paradox, if humans are able to build a conscious machine that overcomes human capabilities: Is the machine more intelligent than humans or are humans still more intelligent because we could build it? The intelligence definition would move again, according to AI successes and new technologies reached.

The ultimate goal of all these discussions is to emphasize that trying to make conscious machines or trying to overcome humans is not the path to improve machines, and indeed, to overcome humans is a contradiction in itself. Futurists speak about super machines with super-human characteristics, but they stimulate these ideas without any care about what means to be a human or even simple, but amazing kind of animals which are still much smarter than computers. To make better machines, science should not focus on anthropocentric presumptions nor compare the intelligence of a machine with human intelligence. The comparison should be according to a general definition of intelligence, as it is stated above. This definition is complex enough and very ambitious goal for any kind of AI. In this way, better machines will be the type 0 and ∞ cognition without anthropomorphic requirements, which will be able to find different solutions to human problems and probably unimaginably better than humans. These machines would be able to imitate some human behavior if needed, but never achieve the genuine social or emotional interaction that humans and animals already have.

On another side, the question about replicating human capabilities is still interesting and important, but for reasons which are not efficient, optimal or better machines. The interest of studying how to implement genuine human features in machines is one academic and even ethical goal, as for example a strategy to avoid animal experimentation. As it was shown above, robots and machines would not be able to replicate the subset of the human being if they do not replicate important features of brains-hardware mentioned previously. These properties are apparently closely connected with important emergent properties which are a fundamental part of consciousness, and some features of consciousness are needed to replicate moral thinking as a crucial and remarkable capability of human beings. That is why, to really understand the biological complexity and mechanisms associated with these emergent properties, the construction of artificial machines based on soft and biological properties/principles can allow us to manipulate and find different kinds of mechanisms until reaching some of the interesting characteristics of living beings. This approach will not take us to more efficient machines, quite the contrary, these machines will be inefficient and if, for instance, type 1 cognition is achieved, they will be closer to some animals, more than good and simple current machines.

That is why, finally, AI could be divided in (1) Biological-Academic Approach, to achieve human intelligence for academic proposes, as for example, instead of using animals to understand consciousness, trying to use robots to implement theories about how consciousness or other important biological features are working. However, once the ultimate goal is reached, for instance, the understanding of consciousness, the knowledge should not be used to replicate or massively produce conscious machines. It would be essentially an ethical question, at the same level or even more intractable than cloning animal issues. (2) Efficient Approach, to make better robots and machines, which can help us with important tasks that are difficult to perform or improve the human performance. The goal is efficiency and performance. In this approach, some principles from biology can be useful, such as modern applications of neural networks, but the final goal would not be to achieve high-level cognition. The implementation in silicon of biological and physical principles of high-level cognition in humans and animals will help us to improve some performances, but these technologies will never replicate truly social interactions, and it should not be expected, because these kinds of interactions are apparently connected with hardware dependences of biological brains. Of course, it is expected to imitate some of them and even incorporate mixed systems between efficient silicon architectures and inefficient soft materials to reach this goal, but any attempt should be conscious of their intrinsic limitations.

## Conclusions

These comments seek to motivate discussion. The first objective was to show typical assumptions and misconceptions when we speak about AI and brains. Perhaps, in sight of some readers, this article is also based on misunderstandings, which would be another evidence of the imperative need for close interaction between biological sciences, such as neuroscience, and computational sciences. The second objective was tried to overcome these assumptions and explore a hypothetical framework to allow conscious machines. However, from this idea emerge paradoxical conclusions of what a conscious machine is and what it implies.

The hypotheses stated above are part of a “proof of concept” to be commented and reformulated. They are part of a work in progress. Thanks to category theory, process theories and others theoretical frameworks, it is expected to develop these ideas on consciousness interaction hypothesis more deeply and relate them with other theories on consciousness, its differences and similarities. In this respect, it is reasonable to consider that a new focus that integrates different theories is needed. This article is just the starting point of a global framework on the foundation of computation, which expects to understand and connect physical properties of the brain with its emergent properties in a replicable and implementable way to AI.

In conclusion, one suggestion of this paper is to interpret the idea of information processing carefully, perhaps in a new way and in opposition to the usual computational meaning of this term, specifically in biological science. Further discussions which expand this and other future concepts are more likely to be fruitful than mere ideas of digital information processing in the brain. Additionally, although this work explicitly denies the analogy brain-digital-computer, it is still admissible a machine-like-brain, where consciousness interaction could be an alternative to implement high intelligence in machines and robots, knowing the limitations of this approach. Even if this alternative is neither deterministic nor controlled, and presents many ethical questions, it is one alternative that might allow us to implement a mechanism for a conscious machine, at least theoretically. If this hypothesis is correct and it is possible to reach the gap of its implementation, any machine with consciousness based on brain dynamics may have high cognitive properties. However, some type of intelligence would be more developed than others, because, by definition, its information processing would also be similar to brains which have these restrictions. Finally, these machines would paradoxically be autonomous in the most human sense of this concept.

## Author contributions

The author confirms being the sole contributor of this work and has approved it for publication.

### Conflict of interest statement

The author declares that the research was conducted in the absence of any commercial or financial relationships that could be construed as a potential conflict of interest.
